# (*S*)-1-Carb­oxy-2-(4-nitro­phen­yl)ethanaminium bromide

**DOI:** 10.1107/S1600536809035211

**Published:** 2009-09-09

**Authors:** Bo Wang

**Affiliations:** a Ordered Matter Science Research Center, College of Chemistry and Chemical Engineering, Southeast University, Nanjing 210096, People’s Republic of China

## Abstract

In the crystal structure of the title compound, C_9_H_11_N_2_O_4_^+^·Br^−^, the ethanaminium cations and Br^−^ anions are linked together by N—H⋯Br and O—H⋯Br hydrogen bonding. In the cation, the nitro group is twisted with respect to the benzene ring, making a dihedral angle of 21.43 (5)°.

## Related literature

For amino acid derivatives as ligands for the construction of metal-organic frameworks, see: Fu *et al.* (2007 ▶).
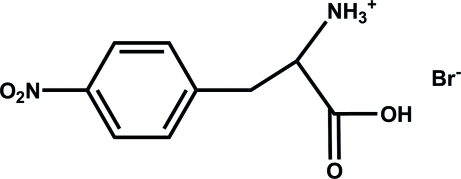


## Experimental

### 

#### Crystal data

C_9_H_11_N_2_O_4_^+^·Br^−^
*M* *_r_* = 291.11
Monoclinic, 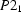

*a* = 5.5378 (11) Å
*b* = 7.4158 (15) Å
*c* = 14.246 (3) Å
β = 91.15 (3)°
*V* = 584.9 (2) Å^3^
*Z* = 2
Mo *K*α radiation
μ = 3.52 mm^−1^
*T* = 298 K
0.40 × 0.05 × 0.05 mm

#### Data collection

Rigaku Mercury2 diffractometer
Absorption correction: multi-scan (*CrystalClear*; Rigaku, 2005 ▶) *T*_min_ = 0.76, *T*_max_ = 0.84
5994 measured reflections
2633 independent reflections
2427 reflections with *I* > 2σ(*I*)
*R*_int_ = 0.039

#### Refinement

*R*[*F* ^2^ > 2σ(*F* ^2^)] = 0.032
*wR*(*F* ^2^) = 0.069
*S* = 1.04
2633 reflections
146 parameters
1 restraint
H-atom parameters constrained
Δρ_max_ = 0.26 e Å^−3^
Δρ_min_ = −0.31 e Å^−3^
Absolute structure: Flack (1983 ▶), 1202 Friedel pairs
Flack parameter: −0.025 (11)

### 

Data collection: *CrystalClear* (Rigaku, 2005 ▶); cell refinement: *CrystalClear*; data reduction: *CrystalClear*; program(s) used to solve structure: *SHELXTL/PC* (Sheldrick, 2008 ▶); program(s) used to refine structure: *SHELXTL/PC*; molecular graphics: *SHELXTL/PC*; software used to prepare material for publication: *SHELXTL/PC*.

## Supplementary Material

Crystal structure: contains datablocks I, global. DOI: 10.1107/S1600536809035211/xu2595sup1.cif

Structure factors: contains datablocks I. DOI: 10.1107/S1600536809035211/xu2595Isup2.hkl

Additional supplementary materials:  crystallographic information; 3D view; checkCIF report

## Figures and Tables

**Table 1:** Hydrogen-bond geometry (Å, °)

*D*—H⋯*A*	*D*—H	H⋯*A*	*D*⋯*A*	*D*—H⋯*A*
N1—H1*A*⋯Br1^i^	0.89	2.54	3.355 (3)	153
N1—H1*B*⋯Br1	0.89	2.46	3.340 (2)	168
N1—H1*C*⋯Br1^ii^	0.89	2.59	3.440 (3)	161
O1—H1⋯Br1^iii^	0.85	2.38	3.174 (3)	155

Symmetry codes: (i) 
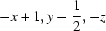
; (ii) 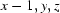; (iii) 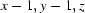.

**Fig. 1.**
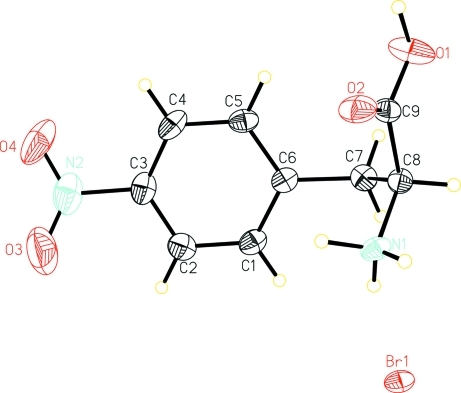
The structure of the title compound with the atomic numbering scheme. Displacement ellipsoids are drawn at the 30% probability level.

## supplementary crystallographic information

### Comment

Amino acid derivatives are a class of excellent ligands for the construction of novel metal-organic frameworks (Fu *et al.*, 2007). We report here the crystal structure of the title compound.

The title compound is built up from a Br^-^ anion and a protonated amino group cation (Fig. 1). The nitro group is twisted from the benzene ring plane by a dihedral angle of 21.43 (5)°, and the 2-aminopropanoate substituent group is a zig-zag chain.

The crystal packing is stabilized by cation-anion N—H···Br and O—H···Br H-bonds building an infinite two-dimensional network developing parallel to the (1 1 0) plane (Table 1).

### Experimental

A mixture of 2-amino-3-phenylpropanoic acid (4.71g, 30 mmol), concentrated nitric acid (4.0 ml, 14 M) and concentrated sulfuric acid (1.5 ml, 18 M) was stirred at 383 K for 3 h under nitrogen atmosphere. The resulting solution was poured into ice water (100 ml), then filtered and washed with distilled water. The crude product was recrystallized with distilled water by adding dilute HBr (4 ml, 4 M) to yield colorless needle-like single crystals.

### Refinement

H atoms were positioned geometrically and treated as riding with C—H = 0.93 (aromatic), 0.97 (methylene), 0.98 Å (methine) and N—H = 0.89 Å, O—H = 0.85 Å with *U*_iso_(H) = 1.2*U*_eq_(C) and *U*_iso_(H) = 1.5*U*_eq_(O,N).

### Crystal data

**Table:**

C_9_H_11_N_2_O_4_^+^·Br^−^	*F*(000) = 292
*M**_r_* = 291.11	*D*_x_ = 1.653 Mg m^−^^3^
Monoclinic, *P*2_1_	Mo *K*α radiation, λ = 0.71073 Å
Hall symbol: P 2yb	Cell parameters from 2427 reflections
*a* = 5.5378 (11) Å	θ = 3.1–27.4°
*b* = 7.4158 (15) Å	µ = 3.52 mm^−^^1^
*c* = 14.246 (3) Å	*T* = 298 K
β = 91.15 (3)°	Needle, colourless
*V* = 584.9 (2) Å^3^	0.40 × 0.05 × 0.05 mm
*Z* = 2	

### Data collection

**Table:**

Rigaku Mercury2 diffractometer	2633 independent reflections
Radiation source: fine-focus sealed tube	2427 reflections with *I* > 2σ(*I*)
graphite	*R*_int_ = 0.039
Detector resolution: 13.6612 pixels mm^-1^	θ_max_ = 27.4°, θ_min_ = 3.1°
CCD profile fitting scans	*h* = −7→7
Absorption correction: multi-scan (*CrystalClear*; Rigaku, 2005)	*k* = −9→9
*T*_min_ = 0.76, *T*_max_ = 0.84	*l* = −18→18
5994 measured reflections	

### Refinement

**Table:**

Refinement on *F*^2^	Secondary atom site location: difference Fourier map
Least-squares matrix: full	Hydrogen site location: inferred from neighbouring sites
*R*[*F*^2^ > 2σ(*F*^2^)] = 0.032	H-atom parameters constrained
*wR*(*F*^2^) = 0.069	*w* = 1/[σ^2^(*F*_o_^2^) + (0.02*P*)^2^] where *P* = (*F*_o_^2^ + 2*F*_c_^2^)/3
*S* = 1.04	(Δ/σ)_max_ < 0.001
2633 reflections	Δρ_max_ = 0.26 e Å^−^^3^
146 parameters	Δρ_min_ = −0.31 e Å^−^^3^
1 restraint	Absolute structure: Flack (1983), 1202 Friedel pairs
Primary atom site location: structure-invariant direct methods	Flack parameter: −0.025 (11)

### Special details

**Table:**

Geometry. All esds (except the esd in the dihedral angle between two l.s. planes) are estimated using the full covariance matrix. The cell esds are taken into account individually in the estimation of esds in distances, angles and torsion angles; correlations between esds in cell parameters are only used when they are defined by crystal symmetry. An approximate (isotropic) treatment of cell esds is used for estimating esds involving l.s. planes.
Refinement. Refinement of F^2^ against ALL reflections. The weighted R-factor wR and goodness of fit S are based on F^2^, conventional R-factors R are based on F, with F set to zero for negative F^2^. The threshold expression of F^2^ > 2sigma(F^2^) is used only for calculating R-factors(gt) etc. and is not relevant to the choice of reflections for refinement. R-factors based on F^2^ are statistically about twice as large as those based on F, and R- factors based on ALL data will be even larger.

### Fractional atomic coordinates and isotropic or equivalent isotropic displacement parameters (Å^2^)

**Table:**

	*x*	*y*	*z*	*U*_iso_*/*U*_eq_	
O2	−0.1236 (3)	0.1957 (5)	0.09374 (15)	0.0526 (5)	
C7	0.3705 (6)	0.3156 (4)	0.2428 (2)	0.0396 (7)	
H7A	0.3926	0.2030	0.2766	0.048*	
H7B	0.5266	0.3746	0.2405	0.048*	
C9	0.0598 (5)	0.1544 (4)	0.1348 (2)	0.0365 (8)	
C8	0.2848 (6)	0.2725 (4)	0.1416 (2)	0.0346 (7)	
H8	0.4158	0.2079	0.1109	0.041*	
C6	0.2007 (5)	0.4342 (4)	0.29749 (19)	0.0361 (7)	
C2	0.0984 (6)	0.7325 (5)	0.3539 (2)	0.0488 (10)	
H2	0.1295	0.8556	0.3573	0.059*	
C4	−0.1471 (7)	0.4776 (6)	0.3932 (2)	0.0488 (10)	
H4	−0.2795	0.4309	0.4242	0.059*	
C3	−0.0974 (6)	0.6591 (5)	0.3970 (2)	0.0474 (10)	
C5	0.0006 (6)	0.3660 (4)	0.3434 (2)	0.0418 (8)	
H5	−0.0333	0.2433	0.3402	0.050*	
C1	0.2497 (6)	0.6173 (5)	0.3048 (2)	0.0427 (8)	
H1D	0.3860	0.6640	0.2765	0.051*	
O4	−0.3908 (5)	0.7160 (9)	0.5059 (2)	0.1119 (15)	
N2	−0.2621 (7)	0.7828 (7)	0.4470 (3)	0.0783 (12)	
O3	−0.2547 (8)	0.9417 (7)	0.4254 (4)	0.1225 (16)	
N1	0.2332 (4)	0.4392 (4)	0.08589 (18)	0.0375 (6)	
H1A	0.2284	0.4121	0.0250	0.056*	
H1B	0.3490	0.5200	0.0971	0.056*	
H1C	0.0915	0.4846	0.1024	0.056*	
O1	0.0979 (5)	−0.0009 (3)	0.1777 (2)	0.0697 (9)	
H1	−0.0290	−0.0651	0.1733	0.104*	
Br1	0.72355 (5)	0.69798 (6)	0.112290 (18)	0.04362 (11)	

### Atomic displacement parameters (Å^2^)

**Table:**

	*U*^11^	*U*^22^	*U*^33^	*U*^12^	*U*^13^	*U*^23^
O2	0.0343 (11)	0.0513 (12)	0.0719 (14)	−0.0139 (17)	−0.0077 (10)	0.010 (2)
C7	0.0327 (17)	0.0424 (18)	0.0434 (19)	−0.0014 (14)	−0.0097 (13)	−0.0022 (14)
C9	0.0373 (16)	0.032 (2)	0.0404 (16)	−0.0075 (12)	−0.0008 (13)	−0.0020 (12)
C8	0.0312 (16)	0.0363 (16)	0.0361 (16)	−0.0054 (12)	−0.0016 (12)	−0.0049 (13)
C6	0.0351 (16)	0.0460 (18)	0.0267 (15)	−0.0042 (14)	−0.0064 (12)	0.0013 (13)
C2	0.060 (2)	0.047 (3)	0.0399 (17)	−0.0022 (17)	−0.0021 (16)	−0.0023 (15)
C4	0.0346 (18)	0.076 (3)	0.036 (2)	−0.0130 (19)	0.0044 (14)	−0.0054 (17)
C3	0.0413 (19)	0.065 (3)	0.0356 (17)	0.0010 (17)	−0.0043 (13)	−0.0170 (16)
C5	0.0438 (19)	0.0447 (19)	0.0368 (18)	−0.0144 (14)	−0.0050 (15)	0.0010 (14)
C1	0.045 (2)	0.052 (2)	0.0310 (17)	−0.0108 (15)	0.0004 (14)	−0.0024 (14)
O4	0.074 (2)	0.156 (4)	0.108 (2)	−0.038 (3)	0.0436 (19)	−0.074 (3)
N2	0.064 (3)	0.099 (3)	0.072 (3)	−0.001 (2)	0.002 (2)	−0.046 (2)
O3	0.123 (4)	0.095 (3)	0.150 (4)	0.034 (3)	0.027 (3)	−0.037 (3)
N1	0.0355 (14)	0.0441 (16)	0.0330 (13)	−0.0151 (12)	0.0001 (10)	−0.0006 (12)
O1	0.072 (2)	0.0427 (15)	0.092 (2)	−0.0215 (13)	−0.0365 (17)	0.0197 (14)
Br1	0.04125 (17)	0.04304 (17)	0.04656 (17)	−0.01590 (17)	0.00035 (11)	0.00473 (18)

### Geometric parameters (Å, °)

**Table:**

O2—C9	1.201 (3)	C2—H2	0.9300
C7—C6	1.515 (4)	C4—C5	1.371 (5)
C7—C8	1.543 (4)	C4—C3	1.375 (6)
C7—H7A	0.9700	C4—H4	0.9300
C7—H7B	0.9700	C3—N2	1.486 (5)
C9—O1	1.318 (4)	C5—H5	0.9300
C9—C8	1.525 (4)	C1—H1D	0.9300
C8—N1	1.493 (4)	O4—N2	1.217 (5)
C8—H8	0.9800	N2—O3	1.219 (6)
C6—C1	1.388 (5)	N1—H1A	0.8900
C6—C5	1.393 (4)	N1—H1B	0.8900
C2—C3	1.369 (5)	N1—H1C	0.8900
C2—C1	1.395 (5)	O1—H1	0.8500
			
C6—C7—C8	114.7 (3)	C5—C4—H4	120.3
C6—C7—H7A	108.6	C3—C4—H4	120.3
C8—C7—H7A	108.6	C2—C3—C4	122.1 (3)
C6—C7—H7B	108.6	C2—C3—N2	117.9 (4)
C8—C7—H7B	108.6	C4—C3—N2	119.9 (4)
H7A—C7—H7B	107.6	C4—C5—C6	120.8 (3)
O2—C9—O1	125.0 (3)	C4—C5—H5	119.6
O2—C9—C8	124.5 (3)	C6—C5—H5	119.6
O1—C9—C8	110.5 (3)	C6—C1—C2	121.2 (3)
N1—C8—C9	107.1 (2)	C6—C1—H1D	119.4
N1—C8—C7	112.2 (2)	C2—C1—H1D	119.4
C9—C8—C7	114.4 (3)	O4—N2—O3	126.2 (5)
N1—C8—H8	107.6	O4—N2—C3	117.0 (5)
C9—C8—H8	107.6	O3—N2—C3	116.8 (4)
C7—C8—H8	107.6	C8—N1—H1A	109.5
C1—C6—C5	118.4 (3)	C8—N1—H1B	109.5
C1—C6—C7	119.0 (3)	H1A—N1—H1B	109.5
C5—C6—C7	122.6 (3)	C8—N1—H1C	109.5
C3—C2—C1	118.0 (3)	H1A—N1—H1C	109.5
C3—C2—H2	121.0	H1B—N1—H1C	109.5
C1—C2—H2	121.0	C9—O1—H1	109.3
C5—C4—C3	119.3 (3)		
			
O2—C9—C8—N1	−1.9 (4)	C5—C4—C3—N2	177.2 (3)
O1—C9—C8—N1	176.0 (3)	C3—C4—C5—C6	0.6 (5)
O2—C9—C8—C7	123.1 (3)	C1—C6—C5—C4	1.2 (5)
O1—C9—C8—C7	−58.9 (4)	C7—C6—C5—C4	179.1 (3)
C6—C7—C8—N1	55.2 (4)	C5—C6—C1—C2	−2.3 (5)
C6—C7—C8—C9	−67.1 (3)	C7—C6—C1—C2	179.6 (3)
C8—C7—C6—C1	−99.1 (3)	C3—C2—C1—C6	1.7 (5)
C8—C7—C6—C5	82.9 (4)	C2—C3—N2—O4	−159.6 (4)
C1—C2—C3—C4	0.2 (5)	C4—C3—N2—O4	21.8 (5)
C1—C2—C3—N2	−178.4 (3)	C2—C3—N2—O3	20.1 (6)
C5—C4—C3—C2	−1.3 (5)	C4—C3—N2—O3	−158.4 (4)

### Hydrogen-bond geometry (Å, °)

**Table:**

*D*—H···*A*	*D*—H	H···*A*	*D*···*A*	*D*—H···*A*
N1—H1A···Br1^i^	0.89	2.54	3.355 (3)	153
N1—H1B···Br1	0.89	2.46	3.340 (2)	168
N1—H1C···Br1^ii^	0.89	2.59	3.440 (3)	161
O1—H1···Br1^iii^	0.85	2.38	3.174 (3)	155

Symmetry codes: (i) −*x*+1, *y*−1/2, −*z*; (ii) *x*−1, *y*, *z*; (iii) *x*−1, *y*−1, *z*.
